# Characterization of collagen response to bone fracture healing using polarization-SHG

**DOI:** 10.1038/s41598-022-21876-z

**Published:** 2022-11-02

**Authors:** Anupama Nair, Shu-Chun Chuang, Yi-Shan Lin, Chung-Hwan Chen, Ting-Chen Fang, Hsiao-Chi Chiu, Chi-Hsiang Lien, Shean-Jen Chen

**Affiliations:** 1 College of Photonics, National Yang Ming Chiao Tung University, Tainan, Taiwan; 2 Orthopaedic Research Center, Kaohsiung Medical University, Kaohsiung, Taiwan; 3 Regeneration Medicine and Cell Therapy Research Center, Kaohsiung Medical University, Kaohsiung, Taiwan; 4 Department of Orthopedics, College of Medicine, Kaohsiung Medical University, Kaohsiung, Taiwan; 5 School of Medicine, Kaohsiung Medical University, Kaohsiung, Taiwan; 6 Department of Adult Reconstruction Surgery, Department of Orthopedics, Kaohsiung Medical University Hospital, Kaohsiung Medical University, Kaohsiung, Taiwan; 7 Department of Orthopedics, Kaohsiung Municipal Ta-Tung Hospital, Kaohsiung Medical University, Kaohsiung, Taiwan; 8 Department of Mechanical Engineering, National United University, Miaoli, Taiwan; 9 Taiwan Instrument Research Institute, National Applied Research Laboratories, Hsinchu, Taiwan

**Keywords:** Biological techniques, Optics and photonics

## Abstract

In this study, we extend on the three parameter analysis approach of utilizing a noninvasive dual-liquid–crystal-based polarization-resolved second harmonic generation (SHG) microscopy to facilitate the quantitative characterization of collagen types I and II in fracture healing tissues. The SHG images under various linear and circular polarization states are analyzed and quantified in terms of the peptide pitch angle (PA), SHG-circular dichroism (CD), and anisotropy parameter (AP). The results show that the collagen PA has a value of 49.26° after 2 weeks of fracture healing (collagen type II domination) and 49.05° after 4 weeks (collagen type I domination). Moreover, the SHG-CD and AP values of the different collagen types differ by 0.05. The change tendencies of the extracted PA, SHG-CD, and AP parameters over the healing time are consistent with the collagen properties of healthy nonfractured bone. Thus, the feasibility of the proposed dual-liquid–crystal-based polarization-SHG method for differentiating between collagen types I and II in bone fracture healing tissue is confirmed.

## Introduction

Bone is a complex material composed of organic and inorganic components such as hydroxyapatite, collagen, proteoglycans, non-collagenous proteins, and water^1^. Among these various components, type I collagen accounts for around 90% of the organic matrix of healthy bone. Bone fracture is common in individuals with osteoporosis or osteogenesis imperfecta, or who routinely perform heavy physical activities^1^. Unlike soft tissues, bone fracture healing is a complicated process involving various factors at the cellular and molecular levels^2^. However, despite the many technological advancements which have been made in understanding the factors responsible for fracture healing, 5–20% of patients still suffer from delayed healing or nonunion in long bone fractures^3^. The regeneration of new bone at the fracture site occurs through two different mechanisms: intramembranous calcification and endochondral bone formation. In the former case, the cells undergo direct osteoblastic differentiation and become calcified without any mediated cartilaginous phase^2^. However, in long bone fracture healing, an initial synthesis of cartilage or callus formation precedes endochondral ossification^3^. Type II collagen in the cartilaginous matrix plays an important role in the initial bone healing stage^4^. However, during endochondral ossification, the extent of type II collagen is replaced by type I collagen, which is secreted by the osteoblasts and becomes the predominant protein in the new bone. The collagen structure is primarily constituted of amino acid triplets consisting of alpha chains rich in Gly–X–Y polypeptides, which link to one another and form a characteristic triple-helical structure^5^. The type I collagen heterotrimer comprises two identical α1 (I) and α2 (II) chains and one α2 (I) chain. By contrast, type II collagen consists of three alpha (I) chains^6^. For both types of collagen, the orientation of the collagen fibrils is crucial to bone strength, and significant changes in the orientation direction are observed with age and under physical loading^7^.

Alteration of the collagen matrix is observed in many bone diseases, including osteogenesis imperfecta (OI), which is characterized by impaired bone quality and increased bone fragility. The main trigger for this bone fragility is a compromised crosslinking of the collagen fibers and defective collagen amendment^8^. Patients with OI are liable to undergo multiple fractures in their lifetimes, often with delayed and nonunion healing^8^. Furthermore, the cartilaginous callus formed during the bone repair often shows prominent changes compared to that in normal healthy individuals^8^. Delayed fracture repair is also commonly observed in osteoporotic fracture patients owing to incomplete bone remodelling^9^. It has been suggested that this delayed bone remodelling is due to the disorganized and erratic nature of the collagen in the repair site of the osteoporotic fracture^9^. Determining the orientation of the collagen fibers is therefore of great interest is gauging the progress of fracture healing.

The most commonly used monitoring methods for bone healing are physical evaluation and radiographic imaging^10^. However, many other techniques are also available, including computed tomography (CT), magnetic resonance imaging (MRI), and a range of microscopic techniques involving histopathological evaluation, such as electron microscopy, optical microscopy, and so on. One such microscopic technique for collagen imaging is second harmonic generation (SHG) microscopy, which has emerged as the gold standard technique for the visualization of 3D collagen fibers at the molecular level with unparalleled contrast and specificity. One of the main advantages of SHG is its ability to image unstained samples at a resolution of just several hundred nanometers to a few microns. SHG has been successfully used to image structural proteins with non-centrosymmetric molecular arrangement^11,12^. SHG probes the nonlinear susceptibility tensor (*χ*^(2)^) of the molecules where the radiated light is equal to exactly one half the excitation wavelength^13^.

Biological tissues, such as collagen, myosin, tendons, and muscles, comprise non-centrosymmetric molecules and are thus effective second harmonic generators since their localized nonuniform molecular structures induce significant changes in the nonlinear susceptibility tensor values. Excitation radiation with ingrained polarization enables the acquisition of detailed quantitative insights into biological tissue samples at the sub-cellular level. As such, its differentiation capabilities go beyond those of the SHG intensity alone. Therefore, integrating polarization state generation techniques with SHG [so-called polarization-resolved second harmonic generation (P-SHG) imaging] has attracted great interest as a means of investigating the detailed (fiber-level) collagen structures of biological tissues. P-SHG imaging has been used in multiple medical disciplines including breast cancer diagnosis^14^, skin irregularities^15^, muscle characterization^16^, osteoarthritis diagnosis^17^, and diseased tissue detection^18^. Several studies have also used P-SHG to characterize the anisotropic responses of tendon, collagen, myosin^19^, and actin thin filaments^20^. In P-SHG imaging, quantification and analysis of the specimen can be performed using various methods, including geometrical and statistical analysis^21^, Fourier transform-based analysis^22^, Mueller matrix decomposition^23^, phasor approach^24^, and grey level co-occurrence matrix (GLCM)-based texture analysis^25^. Several methods for probing the molecular distribution of the collagen fibers in the focal volume have also been proposed, such as anisotropy detection^26^, forward/backward ratio analysis^27^, and circular dichroism (CD) quantification^28^.

The polarization direction of the illumination light must be carefully controlled in a sample-specific direction in order to improve the quality of the imaging results. The required orientation of the polarization light relative to the collagen fibers can be achieved by either rotating the sample while keeping the linear polarization state constant or rotating the linear polarization state of the illumination light while keeping the sample stationary using a motorized half-wave plate (HWP) or a polarizing beam splitter (PBS). However, such systems are prone to elliptical distortion or misalignment due to mechanical rotation.

To overcome these difficulties, this study extends the dual-liquid–crystal-based P-SHG microscopy technique proposed by the present group in a previous study^29^ to facilitate the regulation of polarization conducive to the quantitative characterization of collagen types I and II in bone fracture healing tissue. Here, the state of polarizations are purely voltage dependent for linear polarization as well as circular polarization. While the previous study^29^ characterized the collagen types in self-assembled collagen gels, the present work analyses and quantifies the molecular-level properties of collagen types I and II in real-world pathological samples. Su et al.^30^ previously quantified the relative amount of type I and II collagen in rat-tail tendon and trachea cartilage using the *χ*^2^ tensor ratio. Similarly, Campognola et al. differentiated collagen gel type I from type III using a pixel-based polarization method^31^ and SHG-CD analysis technique^32^. Furthermore, Chaudhary et al.^33^ and Mansfield et al.^34^ both used P-SHG imaging to examine the structural reorganization of collagen in different zones of articular cartilage*.* However, to the best of the current authors’ knowledge, the use of P-SHG to quantify and differentiate between collagen types I and II in bone fracture healing tissue has yet to be reported.

Accordingly, in the present study, the peptide pitch angle (PA), SHG-circular dichroism (CD), and anisotropy parameter (AP) of the collagen distribution in bone fracture healing tissue are evaluated and compared after repair times of 2 weeks and 4 weeks, respectively. Similarly, the articular cartilage morphology with respect to different zones are also analyzed to correlate the collagen type II values to the fracture data. The feasibility of the proposed P-SHG method for differentiating between collagen types I and II in the fracture site is confirmed by correlating the P-SHG quantification results obtained after the different healing times with those obtained for the cortical bone region (collagen type I) and articular cartilage region (collagen type II) of healthy bone control samples. In general, the results show that the pixel-based polarization analysis results obtained by the proposed P-SHG method enable the collagen formation in biological samples to be determined irrespective of the fiber orientation. Consequently, it provides an effective approach for quantifying the role of collagen in the extracellular matrix structure of bone healing tissue and the formation of cartilage and connective tissues.

## Experimental results and discussions

### Tibia bone fracture histology

**Figure 1:**
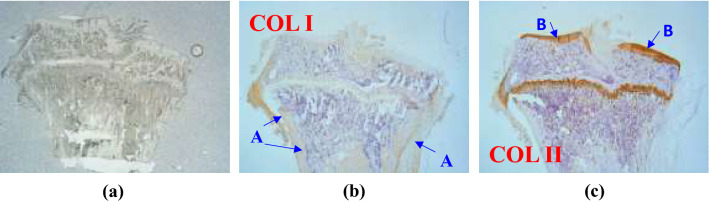
Coronal section of tibia bone: (**a**) bright field image, (**b**) histological image stained for type I collagen (COL I), and (**c**) histological image stained for type II collagen (COL II). Zone A corresponds to cortical bone section rich in collagen type I (brown or brownish-yellow). Zone B corresponds to articular cartilage section rich in collagen type II (dark brown or yellow).

The most common long bone fracture is the tibia bone fracture. Hence, the present research focuses on the cortical bone running through the length of the tibia (rich in collagen type I) and the articular cartilage covering the top surface of the tibia (rich in collagen type II). Figure 1a presents a bright field image of the coronal section of the tibia, while Fig. 1b and c show histological images of the coronal section stained for type I and type II collagen, respectively. Note that in Fig. 1b, Zone A corresponds to cortical bone containing abundant type I collagen, while in Fig. 1c, Zone B corresponds to articular cartilage rich in type II collagen.

### Pitch angle analysis of collagen type I and II morphologies in fracture healing stage

The biochemical, physiological, and mechanical functions of bone are mainly based on the orientation of the collagen structure, which acts as the underlying base of load-bearing tissue. However, the exact mechanisms involved in tissue repair and the role of the collagen fiber orientation in tissue regeneration, are still unclear. Therefore, it is necessary to investigate further into the distribution and fiber orientation of collagen of different types in healing tissue for future application in the development of tissue repair scaffolds. In the present study, the formation of collagen type I and type II in healing bone tissue is quantified by evaluating the peptide PA, SHG-CD, and AP properties of SHG images obtained under various linear and circular polarization states.

**Figure 2:**
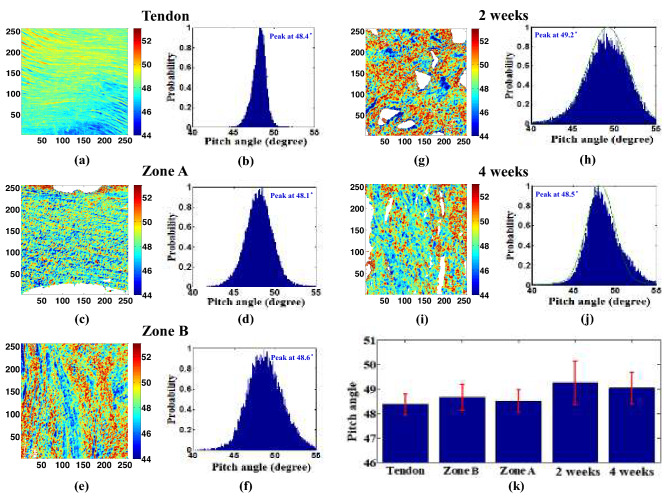
PA maps and corresponding PA histograms for (**a**,**b**) tendon, (**c**,**d**) cortical bone (Zone A), (**e**,**f**) articular cartilage (Zone B) of healthy bone control sample, and (**g**,**h**) bone fracture repair at 2 weeks, and (**i**,**j**) bone fracture repair at 4 weeks. (**k**) Averaged PA values for tendon, articular cartilage (Zone B), cortical bone (Zone A), and fracture repair at 2 weeks, and fracture repair at 4 weeks. Error bars represent standard error of mean. Image size is 50 × 50 μm^2^. The tendon and Zone A (COL I) have lower pitch angle as compared to Zone B (COL II), as observed in the colormaps (**a**,**c**,**e**). Similar decrease in the pitch angle can be observed for the colormap of 4 weeks (**i**) rich in collagen type I as compared to 2 weeks (**g**). The mean values of the healing tissue follow similar trend as that of the control tissue, despite the healing tissues not being significantly different at *p* < 0.05. All the image maps, histograms, and bar graphs were generated by using MATLAB R2019a (Version: 9.6.0.1072779, https://www.mathworks.com/products/new_products/release2019a.html).

The PA was determined for 18 optical cross-sectional images obtained by recording the emission SHG signal while rotating the linear polarization angle through 180° in 10° steps. The resulting PA maps and histograms were then compared with those of a healthy bone control sample. Figure 2a–f show the PA maps and histograms for the tendon, cortical bone (Zone A), and articular cartilage (Zone B) regions of the control bone tissue, respectively. Note that the tendon is included for reference purposes since it is known to be composed almost entirely of type I collagen. Figure 2g–j show the corresponding results for the bone fracture tissue after 2 weeks and 4 weeks of healing, respectively. Finally, Fig. 2k compares the average PA values of the five sets of results. For the control bone tissue, the average peptide PA values of Zone A (rich in collagen type I) and Zone B (rich in collagen type II) are 48.53° and 48.67°, respectively. It is noted that the peptide PA of the cortical bone region (Zone A) is closer to the peptide PA of the tendon (48.38°) than that of the articular cartilage region. After 2 weeks of healing, the average PA of the collagen is 49.26°. However, after 4 weeks, the average PA decreases to 49.05°. The reduction in the average PA is consistent with the results obtained for the control bone tissue, which show that the peptide PA value of collagen type I (which dominates in the later stage of bone tissue healing) is lower than that of collagen type II (which dominates in the initial cartilage or callus formation stage).

### SHG circular dichroism analysis

**Figure 3:**
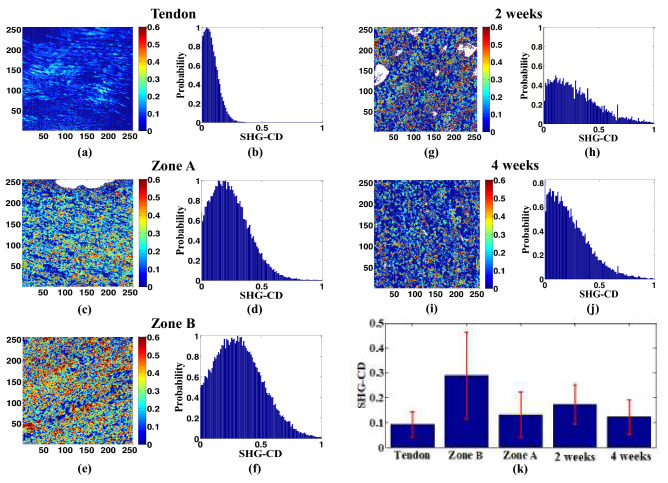
SHG-CD maps and corresponding SHG-CD histograms for (**a**,**b**) tendon, (**c**,**d**) cortical bone (Zone A), (**e**,**f**) articular cartilage (Zone B) of healthy bone control sample, (**g**,**h**) bone fracture repair at 2 weeks, and (**i**,**j**) bone fracture repair at 4 weeks. (**k**) Averaged SHG-CD values for tendon, articular cartilage (Zone B), cortical bone (Zone A), fracture repair at 2 weeks, and fracture repair at 4 weeks. Error bars represent standard error of mean. The image size is 50 × 50 μm^2^. The tendon and Zone A (COL I) have lower SHG-CD value as compared to Zone B (COL II), as observed in the colormaps (**a**,**c**,**e**). Similar decrease in the SHG-CD value can be observed for the colormap of 4 weeks (**i**) rich in collagen type I as compared to 2 weeks (**g**). The healing tissues are significantly different at *p* < 0.05. All the image maps, histograms, and bar graphs were generated by using MATLAB R2019a (Version: 9.6.0.1072779, https://www.mathworks.com/products/new_products/release2019a.html).

The SHG-CD properties of the healing tissue and control bone tissue were obtained by exciting the samples at the same location using right- and left-hand circular polarization lights. SHG-CD analysis is a simplified approach as compared to the PA analysis where a stack of SHG images is required. As shown in Fig. 3, the mean SHG-CD response of the healthy cortical bone (Zone A) is 0.131, while that of the articular cartilage region (Zone B) is 0.289. The mean SHG-CD response of the healing tissue after 2 weeks is 0.172, while that after 4 weeks is 0.122. The reduction in the SHG-CD value with an increasing healing time is again consistent with the SHG-CD response of the control tissue, which shows that the region rich in collagen Type I (Zone A) has a lower SHG-CD value than the region rich in collagen type II (Zone B). The difference in the absolute SHG-CD values of the healing tissue and healthy tissue, respectively, stems most likely from the fact that, during the healing process, both collagen types are present at the same time in certain areas. The present results indicate that the SHG-CD response of collagen types I and II differ by 0.05 for the fracture repair tissue and by 0.16 for the control bone tissue. In other words, the SHG-CD response captures changes in the net chirality of the two collagen types arising from different helical properties in each isoform, and thus provides the means to obtain an exact model of the collagen distribution in fracture repair tissue.

Several previous studies have measured the SHG-CD values of biological tissue surfaces^28,32^. However, to the best of the current authors’ knowledge, the chirality values of collagen types in the same tissue have not been reported and compared. The SHG-CD value is usually found to be higher for irregularly-spaced collagen fibrils^32^. Moreover, the collagen fibrils in the fracture repair zone do not follow a neat pattern, but are irregularly spaced throughout. In addition, the formation of cartilage 2 weeks after fracture and the initiation of bone formation at the repair site both exhibit a patchy pattern with asymmetrical collagen fibers. Accordingly, this study purposely conducted an extended (4-week) study on the collagen type I and type II formation in fracture repair and the resultant analysis values were compared with those of cortical bone and articular cartilage, respectively.

### Molecular anisotropy analysis

**Figure 4:**
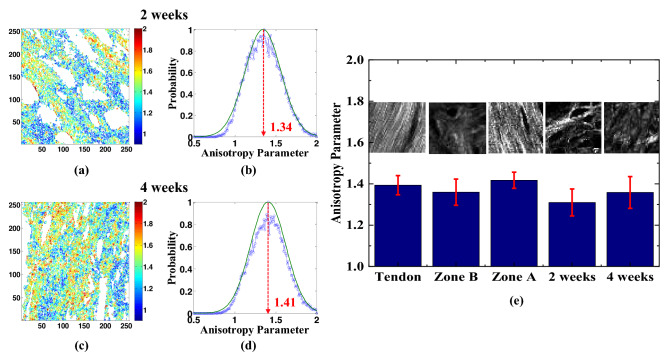
AP maps and corresponding AP histograms for (**a**,**b**) bone fracture repair at 2 weeks and (**c**,**d**) bone fracture repair at 4 weeks. (**e**) Averaged AP values for tendon, articular cartilage (Zone B), cortical bone (Zone A), fracture repair at 2 weeks, and fracture repair at 4 weeks. Error bars represent standard error of mean. The image size is 50 × 50 μm^2^. The bar graph corresponding to the collagen II rich healing tissue at 2 weeks and collagen I rich healing tissue at 4 weeks exhibits a gradual reduction of collagen II along with the simultaneous increase of collagen I. The healing tissues are significantly different at *p* < 0.05. All the image maps, histograms, and bar graphs were generated by using MATLAB R2019a (Version: 9.6.0.1072779, https://www.mathworks.com/products/new_products/release2019a.html).

Articular cartilage is a soft connective tissue composed of only chondrocytes cells and containing primarily type II collagen. As shown in Fig. 4, the average AP value for the tendon in the healthy control sample is around 1.39. By contrast, that for Zone A (cortical bone, rich in collagen type I) is around 1.42, while that for Zone B (articular cartilage, rich in collagen type II) is about 1.36. It is noted that these results are consistent with those reported by the present group in a previous study on the collagen arrangement in gel samples^29^, which showed that collagen type I has a tighter alignment of the collagen fibers than collagen type II and has a higher AP value accordingly. The results presented in Figs. 2 and 3 have indicated that the fracture tissue after 2 weeks of repair is dominated by type II collagen, while that after 4 weeks is dominated by type I collagen. Thus, as shown in Fig. 4e, the average AP value of the healing bone tissue increases from 1.31 at 2 weeks of repair to 1.36 after 4 weeks of repair. The slight discrepancy between the AP values of the healing bone tissue and control bone tissue, respectively, is once again most likely the result of the co-existence of the two different types of collagen in the healing bone tissue. This result demonstrates that our method is sufficiently sensitive to differentiate collagen type I and type II. As such, it provides a feasible approach not only for examining the collagen structure in the cortical bone and articular cartilage regions of the tibia (and other bone tissues in the human body), but also for gauging the state of the tissue repair process following cartilage defect.

### Analyzing collagen in articular cartilage

**Figure 5:**
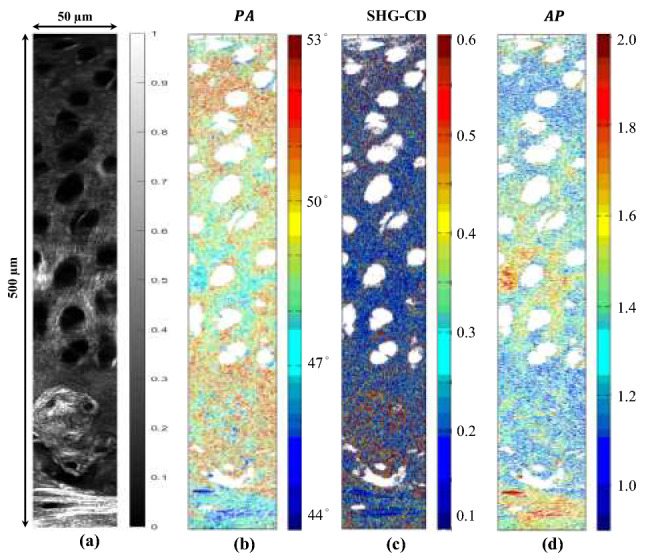
(**a**) SHG intensity, (**b**) PA, (c) SHG-CD, and (**d**) AP image maps at increasing depth of articular cartilage. Size of individual stitched images is 50 × 50 μm^2^. Overall depth imaged is 500 µm. All the image maps were generated by using MATLAB R2019a (Version: 9.6.0.1072779, https://www.mathworks.com/products/new_products/release2019a.html).

**Figure 6:**
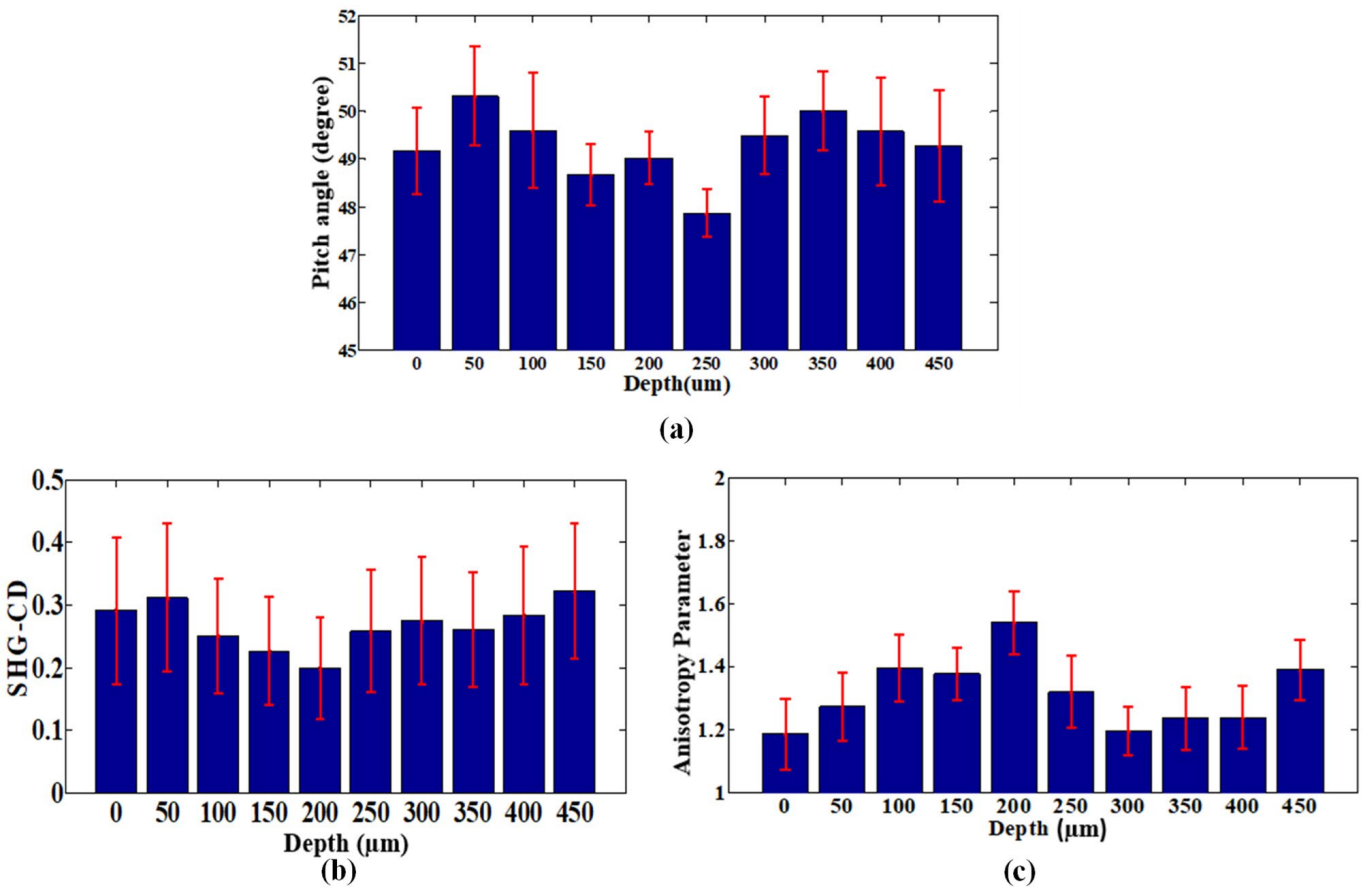
Mean bar graphs for (**a**) PA, (**b**) SHG-CD, and (**c**) AP response of collagen at increasing depth of articular cartilage (0–500 μm). Error bars represent standard error of mean. All the bar graphs were generated by using MATLAB R2019a (Version: 9.6.0.1072779, https://www.mathworks.com/products/new_products/release2019a.html).

Articular cartilage is composed of a superficial zone, a transitional zone, and a deep zone followed by the subchondral bone. The collagen fibril direction in different zones of the cartilage changes with increasing depth into the cartilage, as evidenced by the ratio of forward and backward SHG signal^33^. However, the exact model determining the collagen distribution in articular cartilage is not yet known. Figure 5a shows the SHG imaging results obtained in the present study through a depth of 0–500 μm in the articular cartilage structure. Here, the depths from 0 to 50 µm represents the superficial zone, 50–150 µm represents the transitional zone, followed by the deep zone from 150 to 250 µm. Also, 250–400 µm corresponds to the calcified cartilage area and subchondral bone section is described from 450 µm. The corresponding PA, SHG-CD, and AP maps are shown in Fig. 5b–d, respectively. The mean values of the PA, SHG-CD, and AP at different depths are shown in Fig. 6.

Figure 6a shows that the average peptide PA value reduces gradually from around 49° in the superficial zone to 47° in the deep zone but then increases once again in the calcified cartilage zone to around 50°. Similarly, as shown in Fig. 6b, the SHG-CD value reduces gradually from 0.29 to 0.19 over the depth of 0–200 μm and then increases once again to around 0.28 in the calcified cartilage area. Similarly, the variation in AP values corresponding to distinct depths as shown in Fig. 5d is consistent with different depth where the progression from the superficial zone to the deep zone leads to the increase of values from 1.18 to 1.54. And by going further down to the calcified cartilage zone, a drop in the AP value is observed. Figure 6c shows that, for the present articular cartilage sample, the AP value increases from 1.2 in the superficial zone to around 1.5 at a depth of 200 μm before reducing to approximately 1.23 at a depth of 400 μm. Figure 6a–c show that the peptide PA, SHG-CD, and AP all exhibit a distinct change in the deep zone compared to the other zones. This may be due to the presence of thick collagen fibrils running perpendicular to the articular surface^35^. Subchondral trabecular bone has different mineral phases and collagen distribution than cortical bone^36^. In particular, the cancellous or trabecular bone following the articular cartilage encompasses lamellae with fewer nonreducible crosslinks among the collagen fibers compared to cortical bone, which consists of stable collagen fibrils^37^. Nonetheless, the PA, SHG-CD, and AP values for the subchondral bone region (i.e., for 450–500 μm depth in Fig. 5) are consistent with the previous cortical bone data.

Collagen fibers mostly run parallel to the surface in the superficial zone or tangential layers of articular cartilage. This most probably accounts for the distinctive PA, SHG-CD, and AP values observed in the present study over the depth range of 0–50 μm as compared to the other depths. By contrast, the collagen fibers through the transitional layers run perpendicular to the surface and intersect with one another throughout the middle layer. Although correlating the fiber structure of articular cartilage zones to the callus is difficult owing to the structural and quantity variance at the fracture site, this observation is consistent with the present results, which show that all three analysis mentioned in this study can differentiate the various zones of articular cartilage.

Romijn et al.^38^ used the nonlinear susceptibility tensor to discriminate between the collagen types in cartilage and tendon and showed that the susceptibility ratios were approximated to 1.33 for type I collagen and 1.36 for type II. It is noted that these values are consistent with our present values. However, in their study the collagen types belonged to tendon and cartilage whereas in our study we have tried to differentiate the collagen types in the same repair area.

In the present study, the PA of Zone B, rich in collagen type II, was not significantly higher than that of Zone A, rich in collagen type I of bone tissue. By contrast, in a previous study by the present group on the collagen distribution in gel matrixes^29^, the PA of collagen type II was found to be approximately 2 degrees higher than that of type I. The discrepancy between the two findings may simply reflect the irregular 3D arrangement of collagen structures due to biochemical differences or microenvironment assemblies with varied cell sequences. Similarly, the PA of healing tissue at 2 weeks was not significantly higher than 4 weeks. This could either be due to the presence of both collagen types or due to the spatial heterogeneity in the bone tissue. However, the average PA values at 2 weeks and 4 weeks follow a similar trend as that of the control tissue. Due to the difficulty involved in distinguishing between different collagen types in the same tissue, most previous studies simply quantify the collagen type in different tissues. For example, Campognola’s group^39^ used SHG-CD to distinguish between normal tissue and OI tissue. By contrast, the present study has set out to discriminate between the collagen type (collagen type I and collagen type II) in the same tissue. Furthermore, the SHG-CD signal is not only indicates the molecular chirality, but also performs as a response to tilted collagen molecular orientation relative to the transverse focal plane^40^. Interestingly, the present results for bone tissue show an increase in both the PA and the SHG-CD signal of collagen type II within the probed focal volume. Unfortunately, the present results have shown that an organized collagen structure cannot be identified using a discriminatory approach alone. Furthermore, the present method is unable to determine the exact quantity of collagen of each type in any particular area of the tissue sample. Nonetheless, the method provides the means to identify the specific collagen which dominates at any given time in the tissue repair process and the desmoplastic through the repair process following the collagen type II degradation response.

## Conclusions

The ability to discriminate collagen types in biological samples can facilitate real-time observations of collagen structural changes in pathological processes. However, given the challenges posed in examining multiple molecular forms of collagen structures, little is known about the collagen types and the collagenase during tissue repair. Accordingly, the present study has utilized a dual-liquid–crystal-based P-SHG microscope to image and discriminate between two collagen types (collagen type I and collagen type II) in the fracture repair site of bone tissue. Previous studies have used the AP or susceptibility tensor ratio to study the structural properties of collagen in cartilage or diseased tissue. The present study has additionally performed SHG-CD and peptide PA analyses to substantiate the observation results for the fracture repair tissue. Moreover, the zonal differentiation of articular cartilage evaluated using these analyses method can be ultimately used as a biomarker to study cartilage repair. The results have confirmed that the proposed method enables the reliable differentiation of collagen types I and II in pathological samples and thus provides valuable insights into the real-time structural changes which occur in bone fracture healing tissue.

## Materials and methods

### Experiment animals and tibial fracture model

All of the experimental protocols were conducted in accordance with the “Guide for the Care and Use of Laboratory Animals” of Kaohsiung Medical University and with the approval of the Kaohsiung Medical University Institutional Animal Care and Use Committee (IACUC approval number 97015). Male Sprague–Dawley (SD) rats (350 ± 25 g) at 12 weeks of age were purchased from the National Laboratory Animals Center (Taipei, Taiwan) and housed under standard laboratory conditions (24 °C, 12-h light–dark cycle) with ad libitum food and water.

Twelve 4-month-old male Sprague–Dawley rats were used for the experiments. On being matched for their weights, they were randomly divided into two groups: a control group that received no treatment (*n* = 4) and a fracture healing group with a tibial fracture model bifurcated in to two groups (*n* = 4) for 2 weeks of healing and (*n* = 4) for 4 weeks of healing. The tibial fracture model was implemented using an isolated right tibia fracture with intramedullary needle fixation.

Anesthesia was performed via intraperitoneal injection. Each animal was administered with 87 mg/kg ketamine + 13 mg/kg xyaline, and the right hind limb was prepared and draped to ensure sterile conditions. A longitudinal skin incision with a length of 1 cm was made over the antero-medial aspect of the lower limb. An oscillating saw was then utilized to osteotomize the tibia under continuous irrigation. The fracture was stabilized by inserting a 23-gauge syringe needle into the bone marrow cavity of the tibia. The wound was sutured and covered with sterile dressings for 2 days. The fixation was confirmed to have been properly aligned via radiography.

### Histological sample preparation

The rats were sacrificed for histological analysis and SHG analysis after surgery for the control group and after fracture healing for 2 and 4 weeks. After sacrifice, the right tibiae samples were harvested and fixed in 10% neutral-buffered formaldehyde solution for 2 days. Decalcification of the tibiae was done in 14% ethylenediaminetetraacetic acid (EDTA)/phosphate-buffered saline (PBS) for 14 days. The tibia were then embedded in paraffin and 5-μm microsections were prepared from the coronary plane. The stained tibia samples with marked regions of interest (ROIs) were provided by the medical doctors just for reference. While these stained samples were useful in identifying the fracture ROIs, the imaging was done on unstained tissue sections. Even though some of the structural features were visible in the unstained sample, stained samples with marked ROIs were essential to ensure the preciseness of the ROI chosen. About 20 tissue sections were prepared for each group including Tendon, Zone B, Zone A, fracture repair at 2 weeks, and fracture repair at 4 weeks. For each group, 30 independent ROIs were chosen, where 2 ROIs were from each of the 10 sections, totaling 20 ROIs. For the rest of the 10 sections, since collagen was seemingly scattered among the cortical bone, only one ROI per section was chosen. Hence, a total of 150 ROIs have been examined in this study.

### Immunohistochemically staining for collagen types I and II

5-μm paraffin-embedded slides were deparaffinized in xylene and rehydrated in grade ethanol. For collagen types I and II immunohistochemically staining, the endogenous peroxidase in tissues was firstly blocked by 3% H_2_O_2_, and then the samples were digested by a mixture of 2.5% hyaluronidase and 1 mg/mL pronate at 37 °C for 1 h for epitope retrieval. The slides were then blocked with fetal bovine serum for 1 h. The slides were then incubated using the monoclonal antibody with collagen type I or type II (Chemicon International, Temecula, CA, USA) at 37 °C for 4 h. After the incubation with primary antibody, mouse and rabbit-specific horseradish peroxidase-diaminobenzidine detection immunohistochemically staining kits (Abcam, Cambridge, MA, USA) were used. Finally, sections were counterstained with hematoxylin that showed purple or blue color. The sections appearing brown or brownish-yellow were considered positive staining with collagen type I or type II. Collagen type II has a darker shade as compared to collagen type I.

### Experimental setup

**Figure 7:**
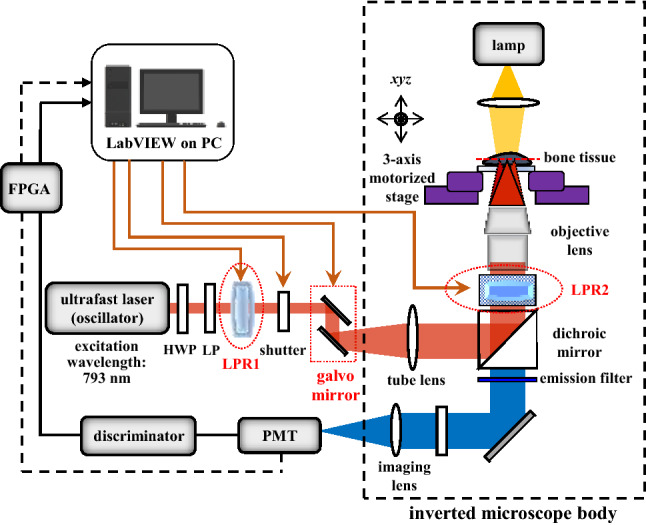
Schematic representation of dual-liquid–crystal-based P-SHG microscope. LPR1 and LPR2 denote first and second liquid crystal polarization rotators, respectively.

SHG imaging was performed using the same optical system as described previously^29^. Briefly, the system comprised an inverted microscope (Axiovert 200, Zeiss Germany) equipped with a mode-locked Ti:sapphire laser as the excitation source (see Fig. 7). The experiments were performed using an excitation wavelength of around 790 nm with an average power of 10 mW at the sample plane, a pulse width of 150 fs, and a repetition rate of 80 MHz. The laser power at the focal plane was controlled using a set of HWPs and a linear polarizer. Image acquisition was performed with an image size of 50 × 50 μm^2^, a field of 256 × 256 pixels, and an exposure time of 1 s. The SHG signal was collected in the forward direction using a bandpass filter set at 390 nm ± 20 nm and a 20×, 0.7 NA objective lens (UPlanApo, Olympus). Besides the inverted microscope and laser source, the other key components in the system included two galvanometer *x* and *y* scanners (6215H, Cambridge, USA), a *z*-axis piezoelectric nano-positioning stage (Nano-F100, Mad City Labs, USA), and a photomultiplier tube (PMT) (H5783P, Hamamatsu, Japan). The collagen structural data were extracted under linear and circular polarization excitation produced using a combination of two liquid crystal polarization rotators (LPRs). The linear polarization lights were obtained by rotating the first liquid crystal rotator to the desired angle by applying the necessary voltage and by placing it in the infinity space of the microscope. Left-hand circular polarization (LHCP) light and right-hand circular polarization (RHCP) light was then produced by rotating the second liquid crystal rotator placed outside the microscope body. Polarization calibration was performed using a tendon sample, with an AP value indicating a complete alignment of the collagen fibers.

### Data analyses

The PA and AP analysis were performed as described previously^29^. Eighteen optical cross-sectional SHG images were obtained by rotating the linear polarization angle through 0°–180° in 10° steps. In general, the nonlinear polarization (*P*) response of a material induced by an incident electric field can be expressed as

1 $$P={\chi }^{\left(1\right)}{E}^{1}+{\chi }^{(2)}{E}^{2}+{\chi }^{(3)}{E}^{3}+\dots ,$$

where *χ*(^*n*^) is the susceptibility tensor which depends on the molecular hyperpolarizability *β*, a measurable quantity that characterizes the strength of the SHG along with its polarization dependence. The PA of the collagen in the present samples was deduced through the combined use of a pixel-based generic model and a single axis molecular model. The pixel-based generic model focuses on the contribution of the SHG tensor components in the focal volume and serves to obtain both the polarization response and the orientation angle^29^. Notably, the generic model is preferred over the molecular model since it allows the randomly-oriented collagen fibers in the focal plane to be distinguished along with the well-aligned fibers. The polarization dependence of the SHG intensity following Fourier decomposition can be expressed as^31,41^

2 $${I}_{SHG }\left(\theta ,{\theta }_{0},r,p,q\right)=r\{\left(5+{p}^{2}+{q}^{2}\right)+4\left(1+p\right)cos2\left(\theta -{\theta }_{0}\right)+2p \, cos4\left(\theta -{\theta }_{0}\right)+4q \, sin2\left(\theta -{\theta }_{0}\right)+2q \, sin4\left(\theta -{\theta }_{0}\right)\},$$

where *θ* is the polarization angle of the incident electric field vector *E*; *θ*_o_ is the equivalent orientation angle from the distribution function expansion; and *r*, *p*, and *q* are three numerical coefficients based on the molecular type and alignment state. In cantrast to the generic model, the single-axis molecular model focuses on the alignment of the dipole moment relative to the physical molecular axis and by considering a cylindrical arrangement of collagen coils where the individual peptide group such as C=O and N–H, provides the peptide pitch angle *θ*_*p*_. The SHG intensity of well-aligned fibers with the focal plane is proportional to the number density of dipoles (*N*) and the square of the second-order polarization, i.e.,

3 $${I}_{SHG }\left(\theta \right)={\left|N{P}^{\left(2\right)}\right|}^{2}=a\left\{{\left(si{n}^{2}\theta +bco{s}^{2}\theta \right)}^{2}+{c}^{2}si{n}^{2}\theta co{s}^{2}\theta \right\},$$

where *a*, *b*, and *c* are numerical coefficients supporting cylindrical and Kleinman symmetry. The helix pitch angle, *α*, is thus obtained by reconstructing the single-axis molecular model from the pixel-based generic model. The anisotropy parameter corresponds to the ratio of tensor elements extracted from the SHG signal. To obtain the anisotropy parameter the intensity parallel and perpendicular to the fiber axis is measured and calculated as following

4 $$b=\sqrt{\frac{{{\chi }^{2}}_{\mathrm{z}zz}}{{{\chi }^{2}}_{zxx}}}=\sqrt{\frac{{I}_{par}}{{I}_{perp}}},$$

where *I*_*par*_ and *I*_*perp*_ are the SHG intensity directions parallel and perpendicular to the fiber orientation, respectively. A normal Gaussian distribution curve is fitted to the orientation angle distribution obtained for every optical section.

The SHG-CD analysis allows the intrinsic chirality of collagen to be detected at the tissular level. The SHG intensity difference resulting from LHCP and RHCP gives rise to SHG-CD which is expressed as

5 $${SHG}_{CD}= \frac{2|({I}_{SHG}\left(RHCP\right)-{I}_{SHG}(LHCP)|}{{I}_{SHG}\left(RHCP\right)+{I}_{SHG}(LHCP)},$$

where ${I}_{SHG}\left(RHCP\right)$ and ${I}_{SHG}\left(LHCP\right)$ are the intensities obtained under right-hand and left-hand circular polarization excitation, respectively. Herein, two cross-polarized SHG images are used to define the orientation of fibers. The scale of difference observed in the intensities under LHCP and RHCP, respectively, was determined from the absolute value summed across the entire field of view since the sign of the SHG-CD response corresponds to the fiber orientation.

All the data in the bar graphs are expressed in mean and standard deviation. A two-sample *t*-test was performed on the average values of each parameter of fracture healing tissue for statistical dissimilarity. The *p* value for statistical significance was set at *p* < 0.05. The two sample *t*-test was performed using Origin 2019b, https://www.originlab.com/.

### Ethics approval

All of the experimental protocols were conducted in accordance with the “Guide for the Care and Use of Laboratory Animals” of Kaohsiung Medical University and with the approval of the Kaohsiung Medical University Institutional Animal Care and Use Committee (IACUC Approval Number 97015). All animal studies in this report is in compliance with the ARRIVE guidelines.

## Data Availability

All of the data generated and analyzed during this study are included in this article.
